# Characterizing *Shigella* species distribution and antimicrobial susceptibility to ciprofloxacin and nalidixic acid in Latin America between 2000–2015

**DOI:** 10.1371/journal.pone.0220445

**Published:** 2019-08-02

**Authors:** Hatim F. Sati, Nienke Bruinsma, Marcelo Galas, Jenny Hsieh, Antonio Sanhueza, Pilar Ramon Pardo, Marcos A. Espinal

**Affiliations:** 1 Antimicrobial Resistance Special Program, Communicable Diseases and Environmental Determinants of Health, Pan American Health Organization, Washington, DC, United States of America; 2 Health Analyses Metrics and Evidence (EIH/HA), Evidence and Intelligence for Action in Health (EIH), Pan American Health Organization, Washington, DC, United States of America; Eugene Lang College of Liberal Arts at the New School, UNITED STATES

## Abstract

**Background:**

Shigellosis is the second leading cause of diarrheal death globally. The global burden has been complicated by the emergence of *Shigella* strains resistant to first line antibiotic treatments such as ciprofloxacin. This study aims to describe the epidemiologic distribution of the most common *Shigella* species, and their antimicrobial susceptibility patterns to ciprofloxacin and nalidixic acid (NA) in Latin America.

**Methods:**

Laboratory data from 19 countries were obtained through the Latin American Network for Antimicrobial Resistance Surveillance (ReLAVRA) from 2000–2015. The Clinical Laboratory Standards Institute reduced susceptibility breakpoints for *Enterobacteriaceae* was used to interpret the disc diffusion tests for *Shigella* susceptibility to ciprofloxacin and NA. Negative binominal regression was used to analyze longitudinal trends of *Shigella* isolates antimicrobial susceptibility.

**Results:**

79,548 *Shigella* isolates were tested and reported between 2000–2015. The most common isolated species were *S*. *flexneri* (49%), and *S*. *sonnei* (28%). There was a steady increase in the proportion of *S*. *sonnei* isolates within the region(p<0.001). The average annual percentage increase (AAPI) in nonsusceptibility was 18.4% (p<0.001) for ciprofloxacin (baseline = 0.3); and 13.2%(p<0.001) for NA (baseline = 3). AAPI nonsusceptibility to ciprofloxacin was 13.3% *for S*. *flexneri* (p<0.04); and 39.9% for *S*. *sonnei* (p<0.001). Honduras, Dominican Republic, Venezuela, and Chile reported the highest increase in nonsusceptibility to ciprofloxacin among all *Shigella* isolates.

**Conclusion:**

There is an increasing trend in *Shigella* nonsusceptibility to ciprofloxacin and NA, including among the most common *shigella* species, in Latin America. This rise of nonsusceptibility among *Shigella* species to commonly used treatments such as ciprofloxacin is alarming and threatens the control and management of this currently treatable infection. Improved data quality, collection and reporting is needed in Latin America to respond effectively to the rising trends observed. This includes the need for quality isolate level epidemiological data; molecular data, and data on antibiotic consumption and use.

## Introduction

Shigellosis is the second leading cause of diarrheal death in the world (164,300 deaths annually, CI: 85 000–278 700), and one of three leading causes of diarrheal deaths in children younger than 5 years [1, 2]. The disease is caused by an *Enterobacteriaceae*, *Shigella*, a facultative anaerobic, non-motile, Gram-negative rod. Infected individuals present with acute invasive enteric infection clinically manifested by watery and sometimes bloody diarrhea [1, 3]. The genus *Shigella* is classified into four main species: *S*. *flexneri*, *S*. *sonnei*, *S*. *boydii*, and *S*. *dysenteriae* [3]. Each of the four-main species cause shigellosis, although vary in terms of virulence and antimicrobial resistance patterns.

The emergence of antibiotic-resistant *Shigella* strains to traditional first-line shigellosis drugs, (ampicillin, trimethoprim-sulfamethoxazole and nalidixic acid) has been a well-recognized public health concern [2, 4, 5]. In response to the increase of antibiotic resistance to these agents, the World Health Organization (WHO) recommended the use of the ciprofloxacin, as alternative empiric antimicrobial treatment for shigellosis [5]. Ciprofloxacin, which was formerly used as a backup drug to treat shigellosis, is currently recommended as the drug of first choice for all patients of all ages presenting with bloody diarrhea; with ceftriaxone and azithromycin as second line treatments [5].

Recently, the isolation of *Shigella* strains resistant to ciprofloxacin has been on the rise [2, 4]. Emergence of resistance has also been reported to other newer alternative drugs such as ceftriaxone, and azithromycin [6, 7]. If this phenomenon continues, shigellosis management and control may be soon undermined. In resource-limited setting, high infectious disease burden and unconstrained access to antimicrobials further complicate shigellosis management and leave fewer viable treatment option.[1, 2, 6]. Therefore, the WHO have included fluoroquinolone-resistance to *Shigella* among the serious antimicrobial resistance (AMR) threats that requires close monitoring alongside urgent research and development of new antibiotics [8, 9].

Reports of increasing resistance to quinolone used for shigellosis in Latin America threaten current shigellosis prevention and control in the region [10]. This study aims to describe the epidemiological distribution of the most common *Shigella* species, their antimicrobial susceptibility patterns and resistance trends to the quinolone’s ciprofloxacin and nalidixic acid, in Latin America.

## Materials and methods

### Data source and description

The Latin American Network for Antimicrobial Resistance Surveillance (ReLAVRA by its Spanish acronym) is a regional antimicrobial resistance surveillance network that was formally established in 1996 by the Pan American Health Organization (PAHO). The network currently constitutes 19 countries, each of which is represented by a national reference laboratory (NRL). For this study, annual aggregate surveillance data for *Shigella* antibiotic susceptibility testing between the years 2000 and 2015 were used. The data were reported to the ReLAVRA network, by the NRL from each of the participating countries (namely: Argentina, Bolivia, Brazil, Chile, Colombia, Costa Rica, Cuba, Ecuador, El Salvador, Guatemala, Honduras, Mexico, Nicaragua, Panama, Paraguay, Peru, Dominican Republic, Uruguay, and Venezuela). Each NRL reported the total number of isolates per year and the overall percentage of resistance (R) and intermediate resistance (I) on data received from laboratories in the country. Each of the NRL is responsible for the external quality control (EQA) for all participating laboratories in that country, routinely ensuring the reliability of the tests performed. This includes the validity of the species identification, and characterization, antibiotic susceptibility tests (ASTs), and data quality.

All *Shigella* isolates reported were recovered from clinical stool specimens and were analyzed by the participating laboratories in each respective country for clinical purposes. This includes identification and disc diffusion testing. All data were then further interpreted and reported to the ReLAVRA network as aggregated values by the receiving NRL. All data were reported in a fully anonymized and de-identified manner.

### Antimicrobial susceptibility testing

Automated methods and the Kirby–Bauer disc diffusion method were used for species identification, and to determine the reduced susceptibility to ciprofloxacin and nalidixic acid [11]. The Clinical and Laboratory Standards Institute (CLSI) reduced susceptibility breakpoints for *Enterobacteriaceae* were used to interpret the disc diffusion tests for *Shigella* susceptibility to ciprofloxacin and nalidixic acid as determined by the CLSI guidance respective of that year [11]. Automated methods for identification of *Enterobacteriaceae* and other aerobic Gram-negative bacteria were done following each local site-specific procedures [12]

The ReLAVRA methodology requires that countries report data of non-duplicated isolates (i.e. patients could only contribute one *Shigella* isolate–per calendar year) [11].

### Statistical analyses

AST data from countries that analyzed and reported less than 30-*Shigella* isolates during a given year were excluded from the analyses for that respective year [11]. Subsequently, for each of the quinolones analyzed (ciprofloxacin and nalidixic acid), the AST results (I) and (R) were grouped together and their sum was referred to as non-susceptible. The overall *Shigella* mean percentage non-susceptible were then calculated for each antibiotic (ciprofloxacin; and nalidixic acid), for each year. The average percentage *Shigella* non-susceptible for the region was then calculated for each antibiotic (ciprofloxacin; and nalidixic acid), for each year. Simple linear regression models were used to assess the direction and significance of changes in the distribution of each species reporting over time.

To account for the variability in the raw data and to assess significance of non-susceptible changes over time, a negative binominal regression model with robust standard errors was used to analyze the changes in antimicrobial non-susceptibility trends over time for each of the antibiotics analyzed (ciprofloxacin and nalidixic acid) reported per calendar year between 2000–2015 [13]. The same analyses were conducted separately for the two most commonly isolated *Shigella* species *S*. *flexneri* and *S*. *sonnei*.

Finally, the average annual percentage variation (increase/decrease) was calculated to describe the overall change in species distribution, and the susceptibility to each antibiotic over time regionally. A significance level of *p<*0.05 was used in these analyses.

All data entry and descriptive analyses were done using Excel software v. 2016 (Microsoft Corp., Redmond, WA), and Tableau software Desktop v. 10.2 (Tableau software Co., Seattle, WA). Inferential statistical analyses were done using Stata v.15.1 (College Station, Texas), and SAS software (release 8.02; SAS Institute Inc., Cary, NC, USA).

## Results

### Descriptive

**Fig 1:**
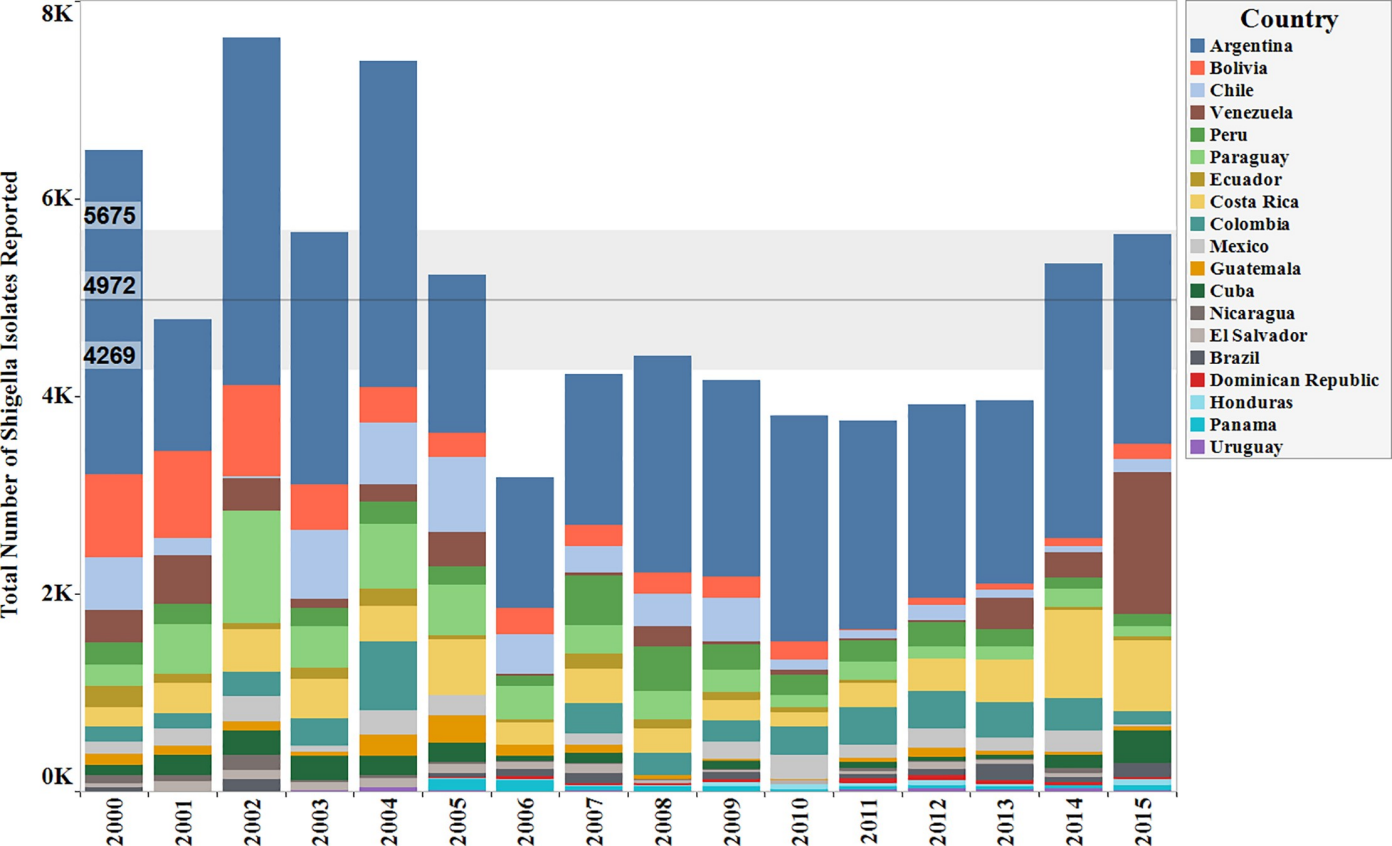
Total number of *Shigella* isolates tested by country, per year (Data aggregates reported by ReLAVRA 2000–2015). The bands represents the average number of isolates reported throughout the time period and the 95% confidence intervals.

Data were available from the 19 countries within the ReLAVRA network. Collectively between the years 2000 and 2015 a total of 79,548 non-duplicated stool isolates for *Shigella* species were reported as tested for antibiotic susceptibility. Within the countries that met the minimal reporting inclusion criteria (of 30 isolates per year), the total numbers of *Shigella* samples reported by each country varied by year. **(Fig 1)**

Across all countries, the average of reported isolates were approximately 4972 cases per year from 2000 to 2015. The maximum and minimum number of isolates reported in a given year were 8368 and 3754 reported in 2004 and 2011 respectively. The average number of isolates reported went from 5442 between 2000 to 2008 to 4,367 between 2009–2015, a 28% decrease (Fig 1). There was an overall decrease in the number of isolates reported throughout the time frame from 2000–2015 (P<0.03).

**Fig 2:**
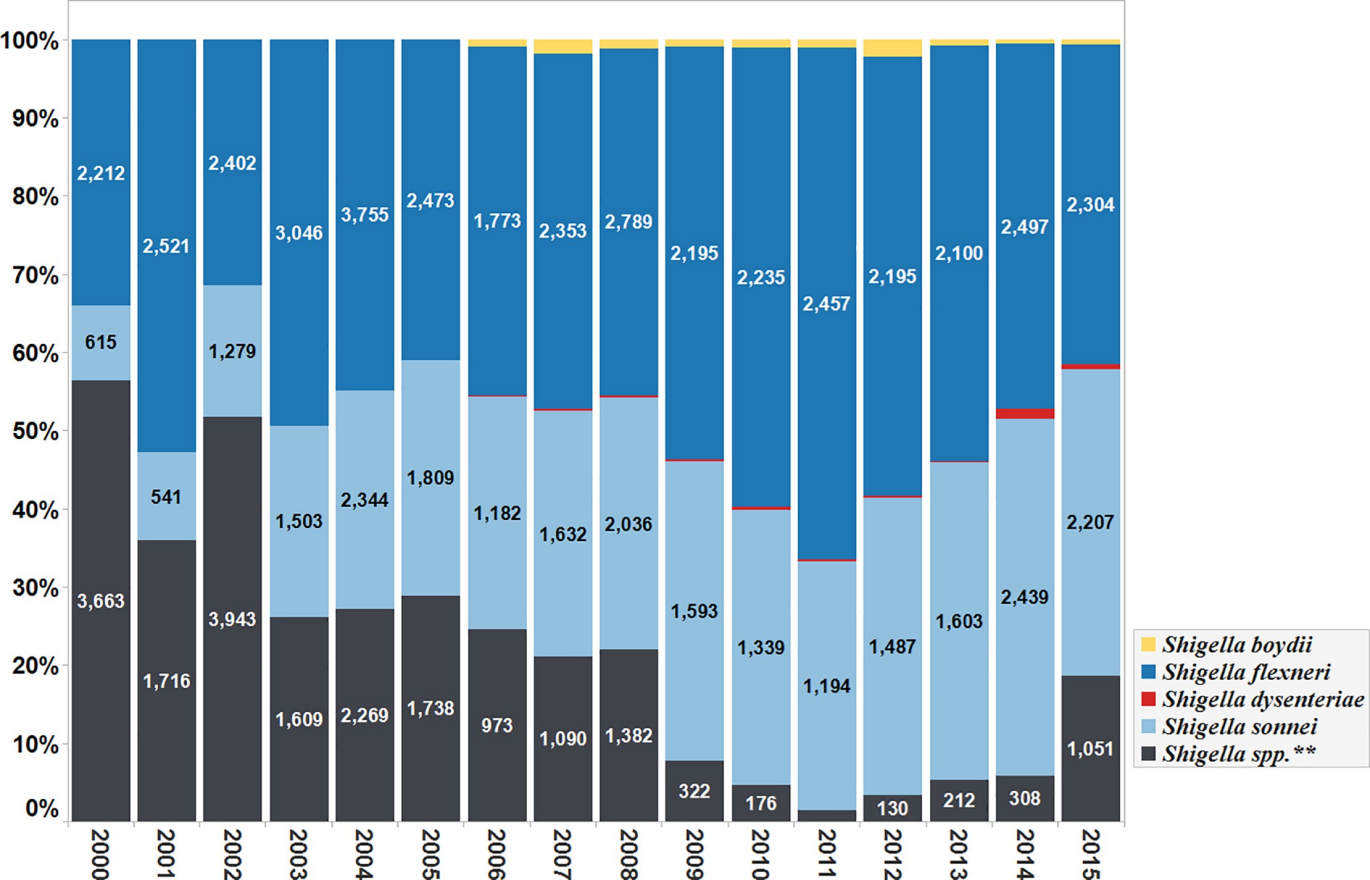
Percentage of total number of *Shigella* species isolated, per year (Data aggregates reported by ReLAVRA 2000–2015).

The most commonly isolated *Shigella* species between the years 2000 to 2015, were *S*. *flexneri* (n = 38,946; 49%), and *S*. *sonnei* (n = 24,477; 28%). Other species identified included *S*. *boydii* (n = 522; 0.7%) and *S*. *dysenteriare* (n = 191; 0.2%). A total of 17,412 (21.9%) isolates were reported without species classification as *Shigella* species (*Shigella* spp.). **(Fig 2)**.

The distribution of the different *Shigella* species reported also varied over the years. Overall, there was a declining trend in the number of isolates reported without species classification (*Shigella* spp.) with an average annual percentage (AAP) decrease of 3% (95% CI: 2.0%-3.9%, p-value < 0.001). The reporting of *S*. *sonnei* isolates increased throughout the time frame with an AAP increase of 2%, (95% CI: 1.5%-2.5%, p-value < 0.001). The reporting of *S*. *flexneri* also increased throughout the time frame with AAP increase of 1% (95% CI: (-)0.1%-2%, p<0.1).

Annual aggregate data for *S*. *boydi*i, and *S*. *dysenteriae* susceptibility testing became available in 2006 and was reported through 2015. (Fig 2). Most of the *S*. *boydii* isolates were reported by Peru, Chile and Cuba. In the same time frame (2006–2015), most of the *S*. *dysenteriae* isolates were reported by Peru, Chile, Cuba and Mexico. The latter reported 41 *S*. *dysenteriae* isolates in 2014.

**Fig 3:**
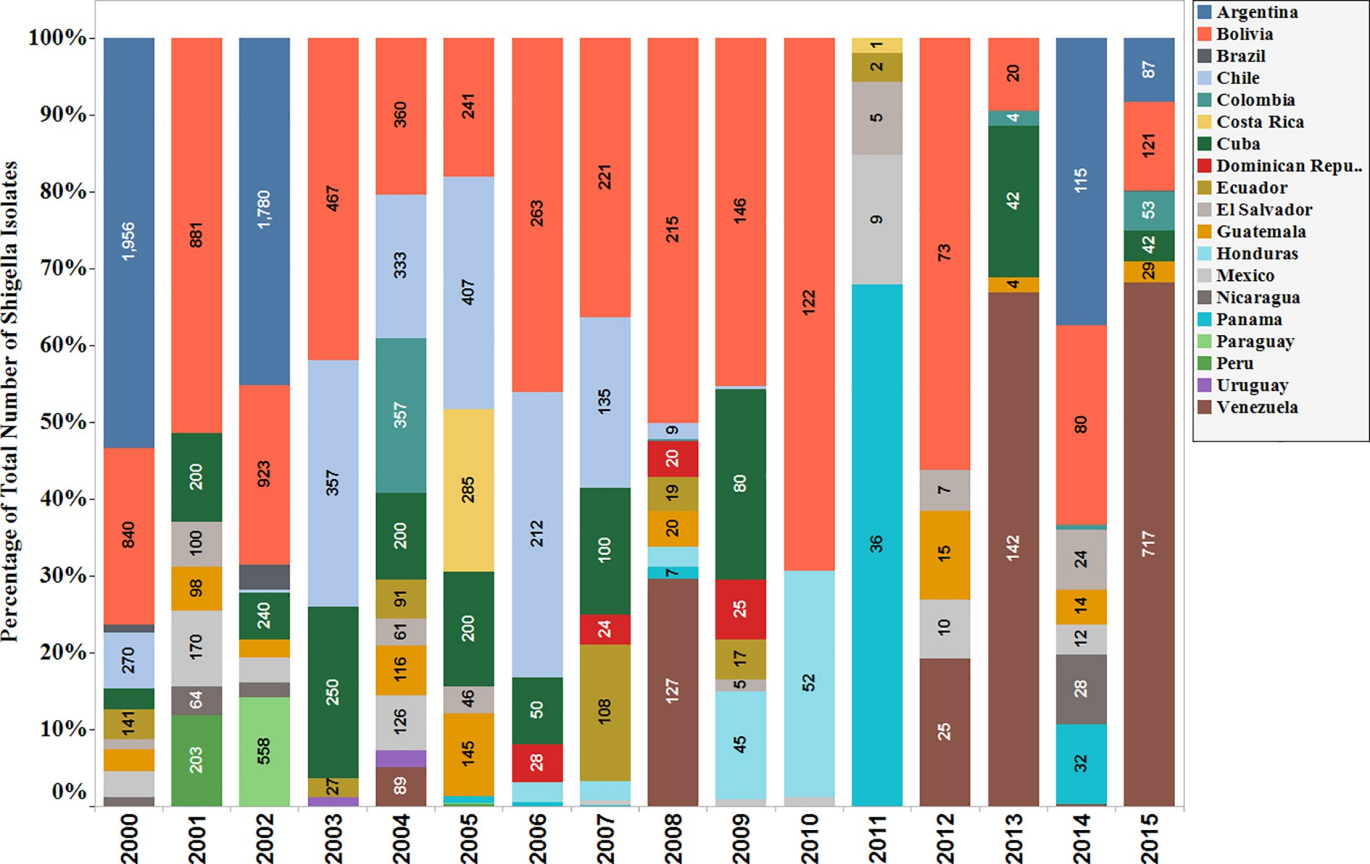
Percentage of total number of *Shigella* species isolated without species classification, per year (Data aggregates reported by ReLAVRA 2000–2015).

The proportion of isolates reported without species classification decreased during the time frame, from 56.4% in 2000 to 18.6% in 2015 (p = 0.0002). It reached its lowest point in 2011 (n = 53 isolates, 1.4%). After 2011 the reporting of *Shigella* isolates without species classification increased slightly in the following years. By the end of the time frame in 2015, the number of *Shigella isolates* without species classification was 1051 isolates (highest since 2011), with most of these isolates reported by Venezuela (68%). **(Fig 3).**

### Susceptibility to ciprofloxacin trend analyses

Between the years 2000–2015 a total of 79,548 *Shigella* isolates were tested for ciprofloxacin susceptibility. 17 countries reported the detection of ciprofloxacin non-susceptible among all *Shigella* isolates tested. Among these, the negative binominal regression analyses showed a statistically significant increasing regional trend in the percentage of non-susceptibility to ciprofloxacin among all *Shigella* isolates. The AAPI was 18.4% per year (95% CI: 10.8%-26.6%, p<0.0001).

**Fig 4:**
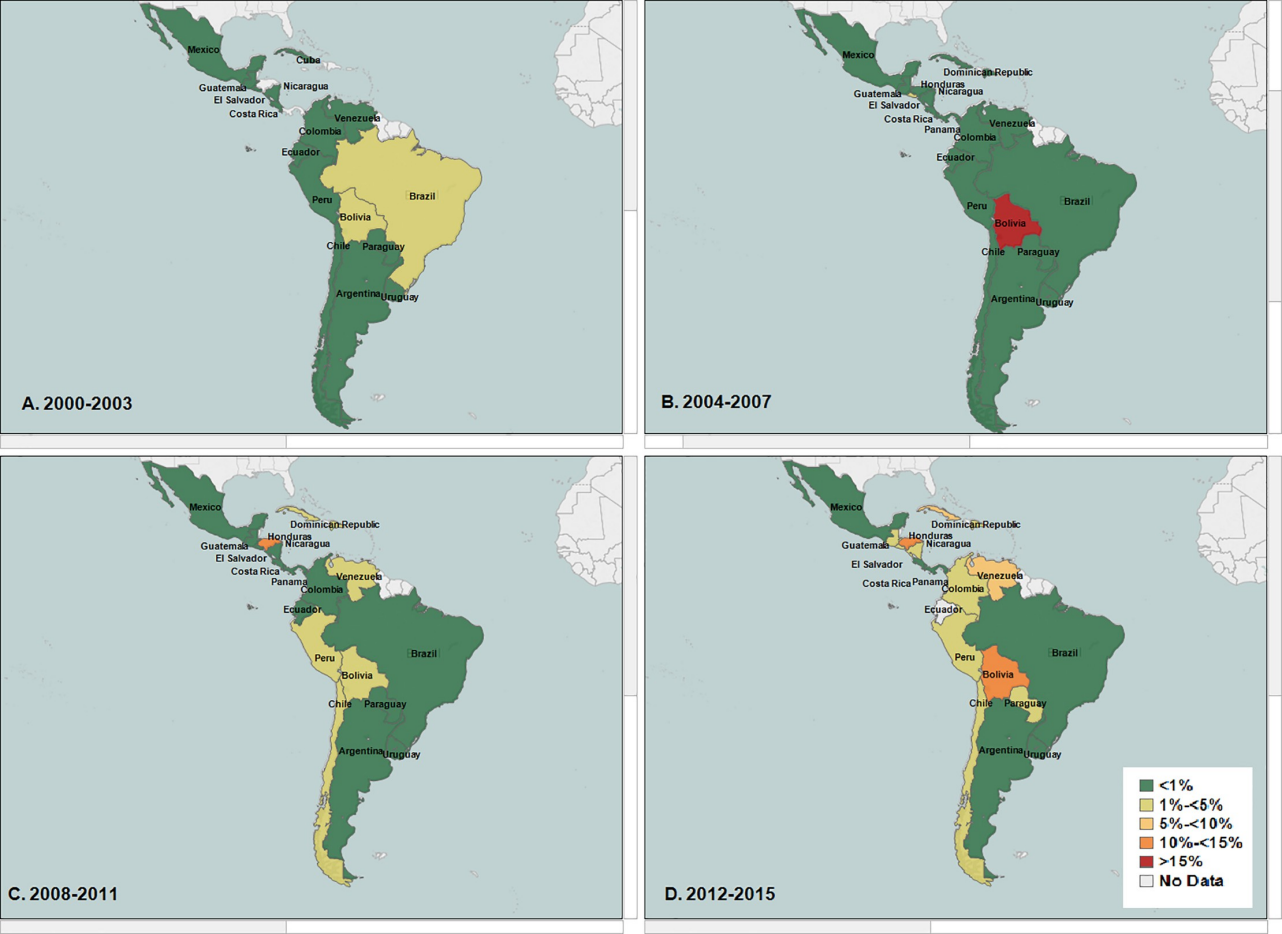
**(A, B, C, D): Average percentage nonsusceptibility All *Shigella* isolates to ciprofloxacin in Latin America (Data aggregates reported by ReLAVRA 2000–2015).** (A) 2000–2003 (B) 2004–2007 (C) 2008–2011 (D) 2012–2015.

Honduras, Dominican Republic, Venezuela, and Chile reported the highest increase in nonsusceptibility to ciprofloxacin among all *Shigella* isolates (nonsusceptibility ranged between 5%-<10%). They were followed by Panama, Cuba, Bolivia, Peru, and Paraguay. **(Fig 4A, 4B, 4C and 4D).**

**Fig 5:**
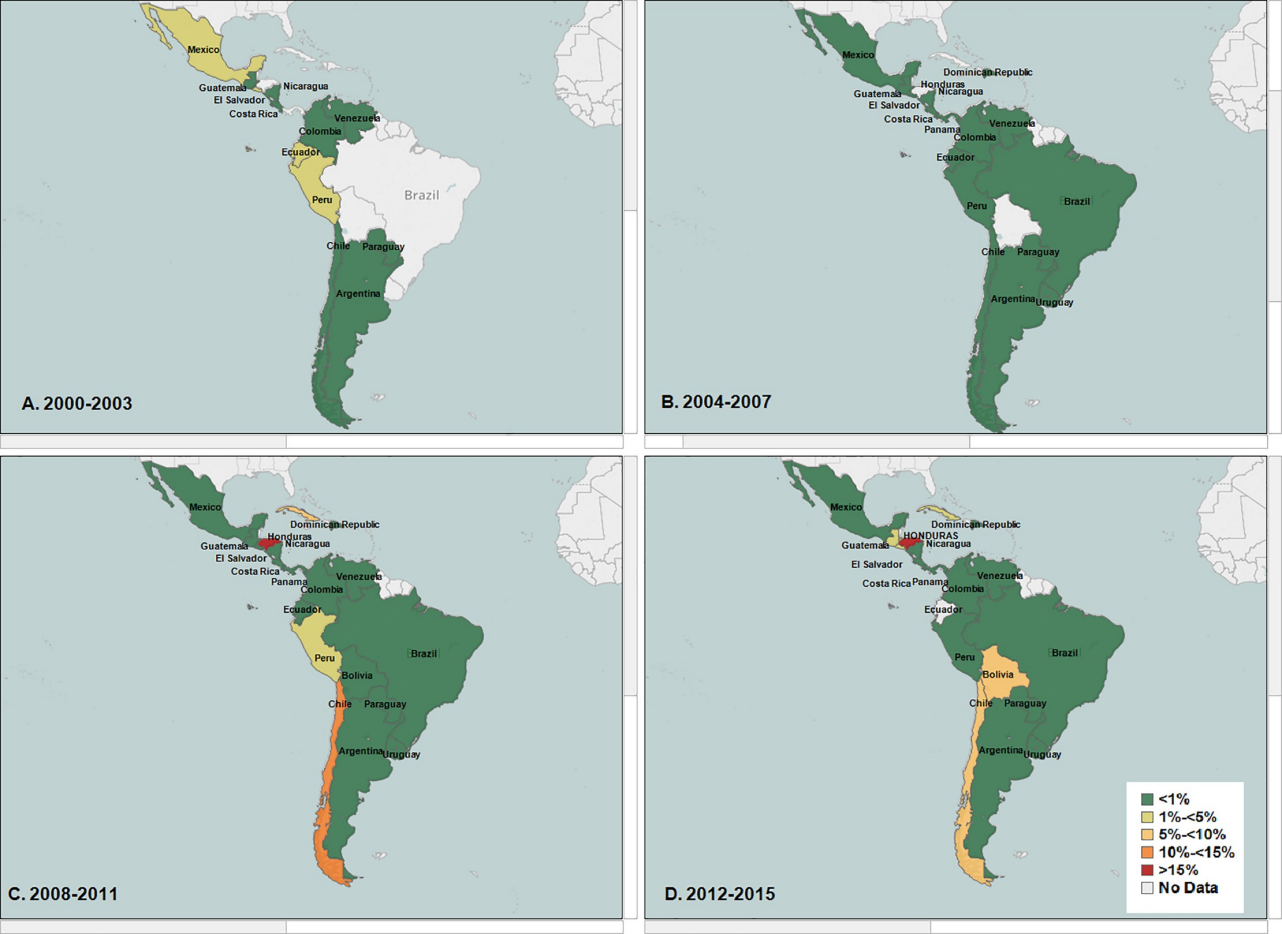
**(A, B, C, D): Average percentage nonsusceptibility among all *Shigella flexneri* isolates to ciprofloxacin in Latin America (Data aggregates reported by ReLAVRA 2000–2015).** (A) 2000–2003 (B) 2004–2007 (C) 2008–2011 (D) 2012–2015.

When stratified by species, similar increasing trends in the regional percentage of non-susceptible isolates to ciprofloxacin was found in the two most common *Shigella* species, *S*. *flexneri* (non-susceptible baseline = 1.0% NS, AAPI = 13.3%; 95% CI: 0.4%-27.9%; p<0.043) and *S*. *sonnei* (non-susceptible baseline = 0, AAPI = 39.9%; 95% CI: 22.1%-60.5%, p<0.001). When analyzed by country this increasing regional trend in the percentage non-susceptibility to ciprofloxacin among all *S*. *flexneri* varied by country, with the highest in Chile (non-susceptible ranged between 5%-<10%), followed by Cuba, Brazil and Peru **(Fig 5A, 5B, 5C and 5D)**.

**Fig 6:**
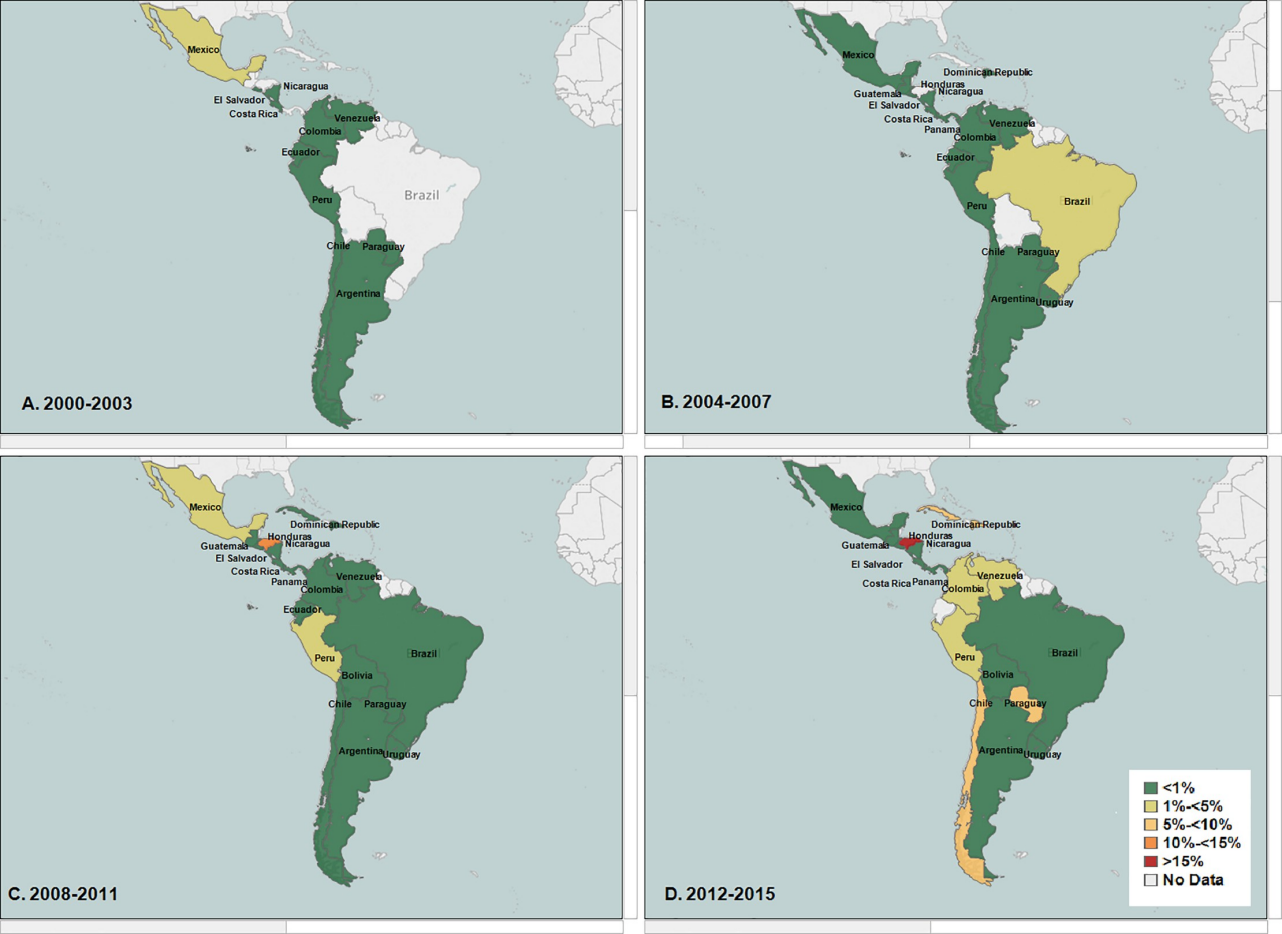
**(A, B, C, D): Average percentage NS among all *Shigella sonnei* isolates to ciprofloxacin in Latin America (Data aggregates reported by ReLAVRA 2000–2015).** (A) 2000–2003 (B) 2004–2007 (C) 2008–2011 (D) 2012–2015.

For *S*. *sonnie*. this increase was highest in Honduras (non-susceptible ranged between 10%-<15%), followed by Dominican Republic, Paraguay (non-susceptible ranged between 5%-<10%). **(Fig 6A, 6B, 6C and 6D)**.

### Susceptibility to nalidixic acid

Between the years 2000–2015, 79,548 *Shigella* isolates were tested for nalidixic acid resistance. Overall, nalidixic acid non-susceptibility was detected in 18 countries. A statistically significant increasing trend was observed in the percentage non-susceptible to nalidixic acid among all *Shigella* isolates during the period of study, with average annual percentage increase (AAPI) of 13.2% (non-susceptible baseline = 3% CI: 7.8%-18.9%, p<0.001). When stratified by species, a significant increasing trend in the percentage non-susceptible to nalidixic acid was found *S*. *sonnei* (non-susceptible baseline = 0.0%, AAPI = 31.7%; CI: 44.8%-19.7%, p<0.001. This increasing trend was none-statistically significant for *S*. *flexneri* (non-susceptible baseline = 1% AAPI = 1.5%; CI: 6.1%-9.8%; p<0.7).

## Discussion

### Epidemiologic distribution of common *Shigella* species

Globally, the most prevalent *Shigella* species are *S*. *flexneri* followed by *S*. *sonnei*, and they account for most of *Shigell*a incidence worldwide outside of an outbreak setting [2, 3]. It has been established that *S*. *flexneri* is common in low and middle-income countries (LMIC) while the latter is more prevalent in high income countries [14]. This epidemiologic distribution may be due to number of interplaying immunologic, virulence, and environmental pressure factors [15]. During the period under review, *S*. *flexneri* was the most commonly reported isolated species followed by *S*. *sonnei*. All the countries in this study are either low or middle-income countries, according to the World Bank country classification [16]. Hence, the findings of this study support this existing supposition.

This study showed a steady significant increase in the proportion of *S*. *sonnei* isolates within the region throughout the time frame. In recent years a number of epidemiological studies have noted similar shifts in the prevalence of *S*. *sonnei* and *S*. *flexneri* [6, 14]. Several complex and intersecting factors may explain this pattern [17]. The surge in the isolation of *S*. *sonnei* was mostly reported in countries and regions that underwent rapid industrialization and water sanitation development [15]. This pertains to Latin America, where the emergence of new economies and growing industrialization, were associated with expansion in water sanitation infrastructure [18]. *S*. *sonnei* isolation in response to better sanitation is not fully understood. One possible explanation is the natural cross-protective immunity to *S*. *sonnei*, induced by natural infection with *Plesiomonas shigelloides*, a Gram-negative *Enterobacteriaceae* common in resource-limited settings, with poor water sanitation [19]. Populations living in such settings maybe exposed to *P*. *shigelloides*, due to contaminated water supplies, and could as a result acquire natural immunity to *S*. *sonnei* [19]. These influences, although uneven, might have led to major shifts in infectious disease patterns and epidemiological conditions that could further explain the observed shift in the prevalence of *S*. *sonnei*. Conversely, there is potential that the observed increase in *S*. *sonnei* may be due to increased capacity for speciation and reporting among countries in the region, especially given the decrease in the number of non-speciated *Shigella* isolates reported in the most recent years.

The prevalence of the other less common species (*S*. *boydii* and *S*. *dysenteriae)* was overall low in this study. This is expected since *S*. *boydii* is commonly reported in Southeast Asia and rarely occurs outside of this region. *S*. *dysenteriae* is more common in outbreak settings associated with civil unrest and refugee crisis [20, 21]. Nevertheless, the increase in reporting of the latter by some countries (e.g. Venezuela) might have some serious implications given the current situation in the some of these countries and calls for further investigation.

### *Shigella* susceptibility to ciprofloxacin and nalidixic acid

The overall *Shigella* non-susceptible to both quinolones (ciprofloxacin, and nalidixic acid) has increased significantly within the region during the 15-year surveillance timeframe. This was the case across all species, as-well-as for the two most commonly isolated *Shigella* species, *S*. *flexneri* and *S*. *sonnei*, and was more pronounced between 2009 and 2015. These results are in accordance with findings in previous reports, which indicated that the emergence of resistance to fluoroquinolones—namely, ciprofloxacin and nalidixic acid—in *Shigella* species is increasing globally [22–24].

A recent systematic review reported increasing patterns in *Shigella* resistance to ciprofloxacin and nalidixic acid in Asia, Africa and, to a lesser degree, in Europe and the Americas [25]. In Latin America, recent publications have reported the emergence of ciprofloxacin resistant isolates in the region [26, 27]. In April of 2017, The United States Center for Disease Prevention and Control outlined some recommendations for management of such *Shigella* isolates with reduced ciprofloxacin susceptibility, in response to the rise of *Shigella* strains with reduced ciprofloxacin susceptibility [28].

The increasing trend of *Shigella* non-susceptible to ciprofloxacin, reported in this study and others, might be due to number of factors. First, the rapid ability of *Shigella* to develop AMR rendered previous first-line shigellosis treatments, such as ampicillin and trimethoprim-sulfamethoxazole, ineffective and shifted treatment recommendations worldwide to alternative drugs (e.g. ciprofloxacin, azithromycin, and ceftriaxone) [29, 30]. Since 2005, ciprofloxacin has been recommended by the WHO as the empiric treatment of first choice for shigellosis, increasing its use for infectious diarrhea globally [4, 8]. Additionally, despite the reported rise in AMR to ciprofloxacin, the drug remains widely used in the empiric and prophylactic management of many other community-acquired infections [31–34]. This all may have driven ciprofloxacin’s empiric use in the region.

Second, in Latin America, empiric prescriptions occur mostly at the community level, and are often not corroborated by clinical microbiology laboratories [35, 36]. In fact, many countries in the region lack enforced regulation for prescription drugs, and antibiotics are readily available over the counter. The unheeding prescription of these agents, combined with lack of regulations for antibiotic use, self-medication, and the high burden of infectious disease may have further stimulated the selection and emergence of ciprofloxacin resistant *Shigella* strains in the region [37]. Nevertheless, the demonstration of an overuse driven selection of resistant strains might be difficult, due to scarcity of antibiotic use data in the region, and the lack of reliable public consumption data.

Finally, *Shigella* possesses a high capacity for rapidly acquiring antibiotic resistances elements. This is specifically true to *S*. *sonnei*, which can acquire resistance genes from other *Enterobacteriaceae* through horizontal gene transfer [38]. This maybe contributed to the observed higher increase in *S*. *sonnei* non-susceptible to ciprofloxacin compare to that of *S*. *flexneri* (AAPI of 39, 9% Vs.13.3% respectively).

The increasing frequency of international travel; demographic and socioeconomic changes in the region, genetic factors, such as cross resistance, might have further facilitated the genetic exchange of resistant elements, and further exacerbated resistant mutant selection and spread [39–41].

In the case of nalidixic acid, over-use-driven *Shigella* resistance to this drug has been reported for decades in LMIC [25, 42]. Nalidixic acid-resistant *Shigella* strains with cross-resistance to other quinolones (including ciprofloxacin) were reported in areas where nalidixic acid was used as the first-choice drug for management [43]. Nalidixic acid use in the empiric treatment of shigellosis has been also been limited in many parts of the world, after evidence emerged that it might be associated with quinolone-induced arthropathy [8, 25, 38]. Nevertheless, because of cross-resistance, nalidixic acid non-susceptibility can be a good indicator for ciprofloxacin resistance [7, 44, 45]. The observed increase in the non-susceptible to nalidixic acid in this study may be an indication of an imminent emergency and propagation of full ciprofloxacin resistance in the region.

## Limitations

This study has several limitations that should be considered. The results are based on the countries analyses of aggregated surveillance data reported by countries, and do not include samples that were not reported to the ReLAVRA network. The susceptibility results (values of I & R) reported are based on CLSI breakpoints corresponding to the year that data were reported and may result in some biases. Considering the updated *Shigella* breakpoints of 2019 for ciprofloxacin, the reported percentage non-susceptibility may be underestimated in the current analyses [11].

Further, these data do not include self-limiting and self-medicated shigellosis cases as most of these cases often go unreported. It is likely that most of the isolates and analyzed clinical samples represent severe and/or treatment-failure, shigellosis cases (which might be associated with MDR strains).

Additionally, the frequency of reporting tested *Shigella* isolates varied between countries, with most countries reporting annually and few countries reported irregularly. There was no information about the geographic and demographic representativeness including patients’ ages and gender or travel history. Therefore, inferences regarding risk groups, key populations and demographic distribution of resistance and serogroups in the region are limited and calls for improvement in the surveillance data in the coming years.

## Conclusion

This study describes an increasing trend in *Shigella* nonsusceptibility to ciprofloxacin and nalidixic acid, including among the most common *Shigella* species, in Latin America. This rise of antibiotic resistance among *Shigella* species to commonly used treatments such as fluoroquinolones, is alarming and it threatens the ability to control and manage this currently treatable infection. The ability to respond quickly to the changing trends in *Shigella* species epidemiologic distribution and resistance patterns call for speedy implementation of prevention and control measures against *Shigella* in Latin American. Proactive approach to AMR surveillance and monitoring is vital for mitigation of further development of resistance. Further, there is a need for improved data quality, collection and reporting, to respond effectively to the epidemiological trends observed, and to better understand the burden of *Shigella* resistance to quinolones in Latin America. This includes the need for quality isolate level molecular and epidemiological data; and data on antibiotic consumption and use in all healthcare sectors. National health authorities’ commitment in the region, as well as operational coordination between national and regional laboratories, and healthcare facilities is needed to inform effective treatment practices, guidelines and control measures nationally and globally.

## Supporting information

S1 Fig Percentage of non-susceptibility in *Shigella isolates* to nalidixic acid & ciprofloxacin in Latin America (Data source: ReLAVRA 2000–2015). ReLAVRA = La Red Latinoamericana de Vigilancia de la Resistencia a los Antimicrobianos.
(TIF)

S2 Fig Percentage of non-susceptibility in *Shigella flexeneri* isolates to nalidixic acid & ciprofloxacin in Latin America (Data source: ReLAVRA 2000–2015). ReLAVRA = La Red Latinoamericana de Vigilancia de la Resistencia a los Antimicrobianos.
(TIF)

S3 Fig Percentage of nonsusceptibility in *Shigella sonnei* isolates to nalidixic acid & ciprofloxacin in Latin America (Data source: ReLAVRA 2000–2015). ReLAVRA = La Red Latinoamericana de Vigilancia de la Resistencia a los Antimicrobianos.
(TIF)

S4 Fig Percentage of total number of *Shigella boydii* isolates tested by country, per year in Latin America (Data source: ReLAVRA 2006–2015). ReLAVRA = La Red Latinoamericana de Vigilancia de la Resistencia a los Antimicrobianos. N = number of isolates reported.
(TIF)

S5 Fig Percentage of total number of *Shigella dysenteriae* isolates tested by country, per year in Latin America (Data source: ReLAVRA 2006–2015). ReLAVRA = La Red Latinoamericana de Vigilancia de la Resistencia a los Antimicrobianos. N = number of isolates reported.
(TIF)

S1 Dataset Dataset_ReLAVRA-Shigella 2000–2015. (XLSX)
