# Supplementary figures and images for: Characterizing Shigella species distribution and antimicrobial susceptibility to ciprofloxacin and nalidixic acid in Latin America between 2000–2015
Source: PLoS One. 2019 Aug 2;14(8):e0220445. doi: 10.1371/journal.pone.0220445 (PMC6677304; doi:10.1371/journal.pone.0220445)

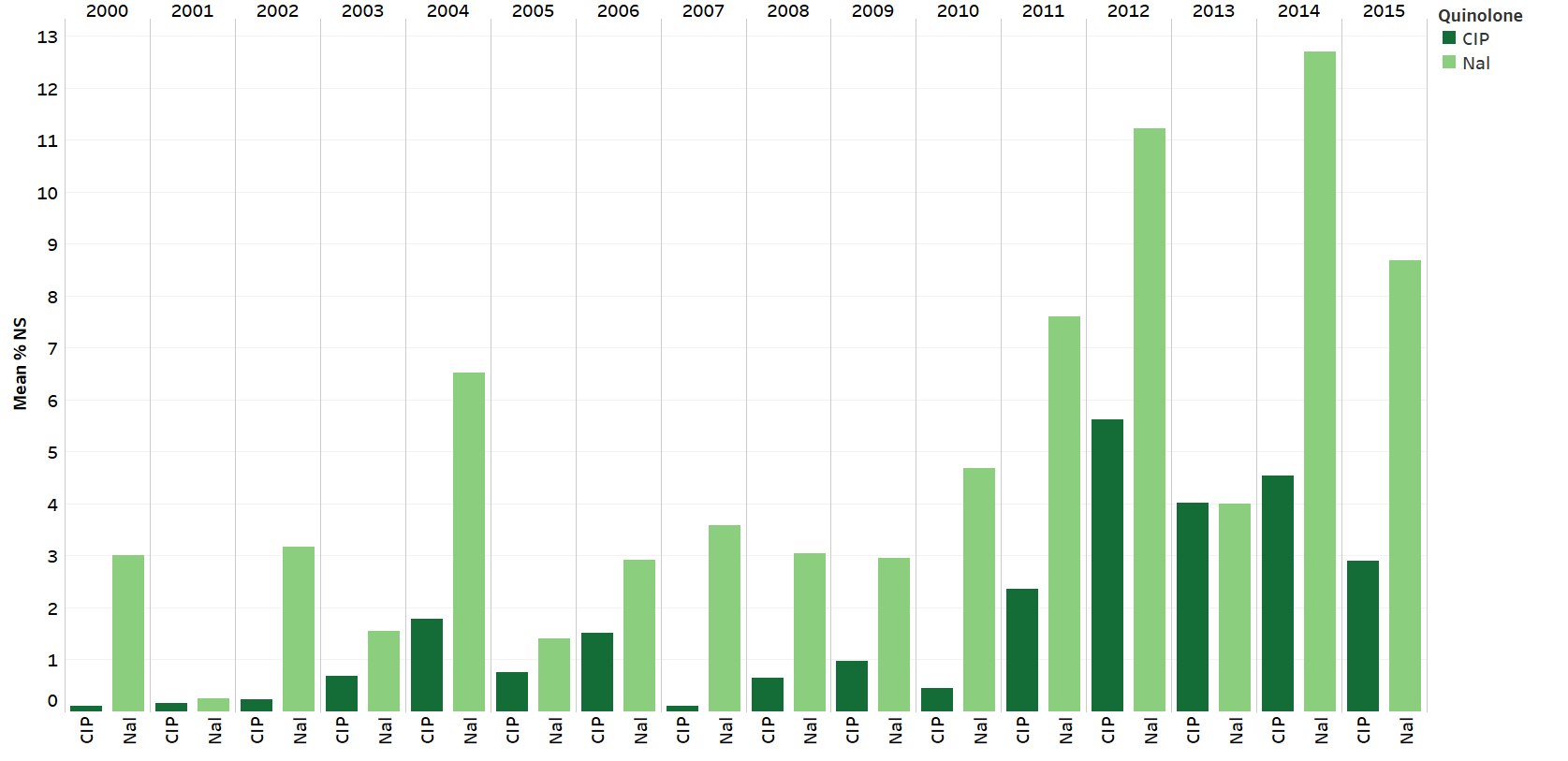

Supplement: S1 Fig — ReLAVRA = La Red Latinoamericana de Vigilancia de la Resistencia a los Antimicrobianos. (TIF) [file pone.0220445.s001.tif]

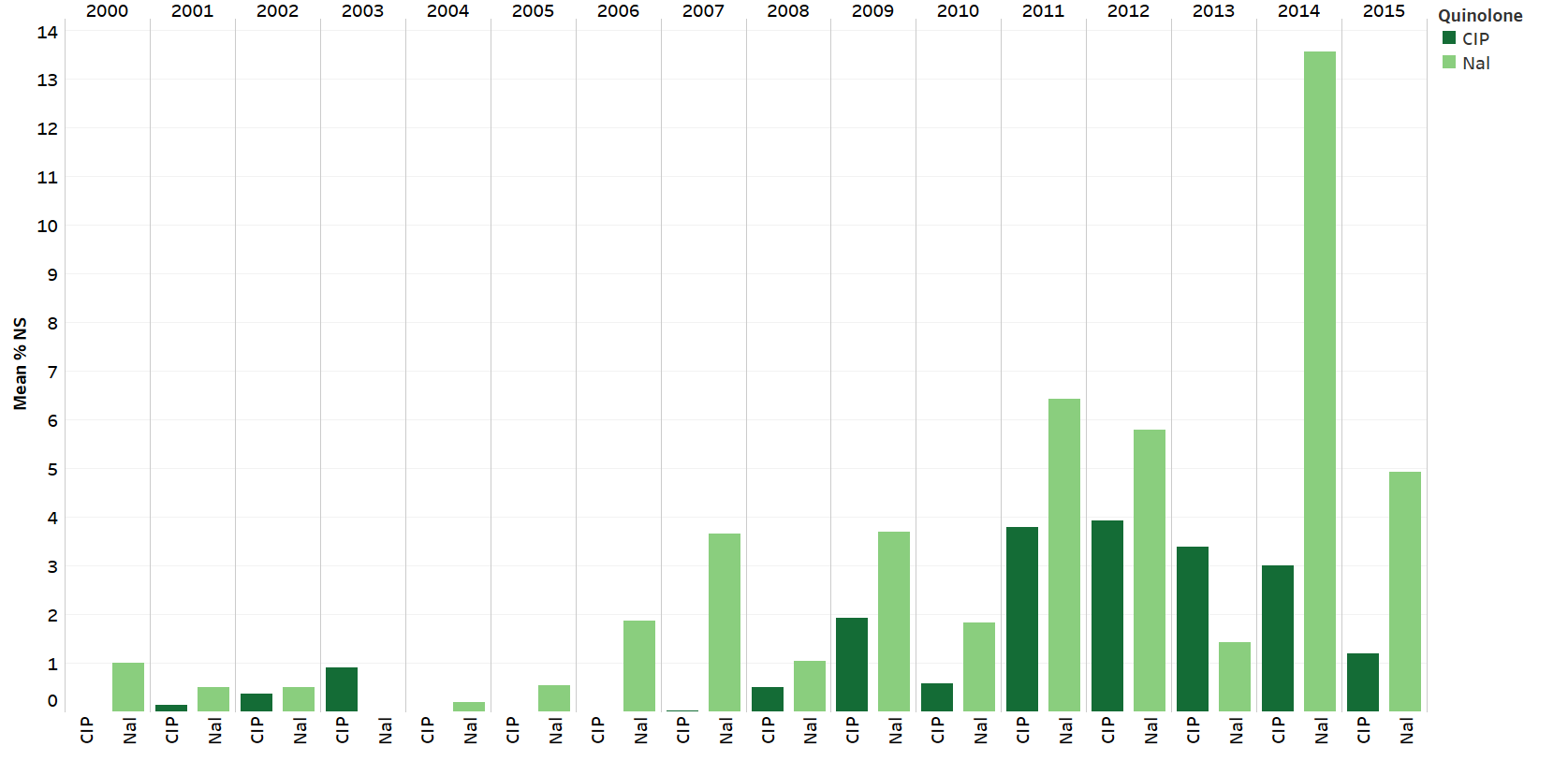

Supplement: S2 Fig — ReLAVRA = La Red Latinoamericana de Vigilancia de la Resistencia a los Antimicrobianos. (TIF) [file pone.0220445.s002.tif]

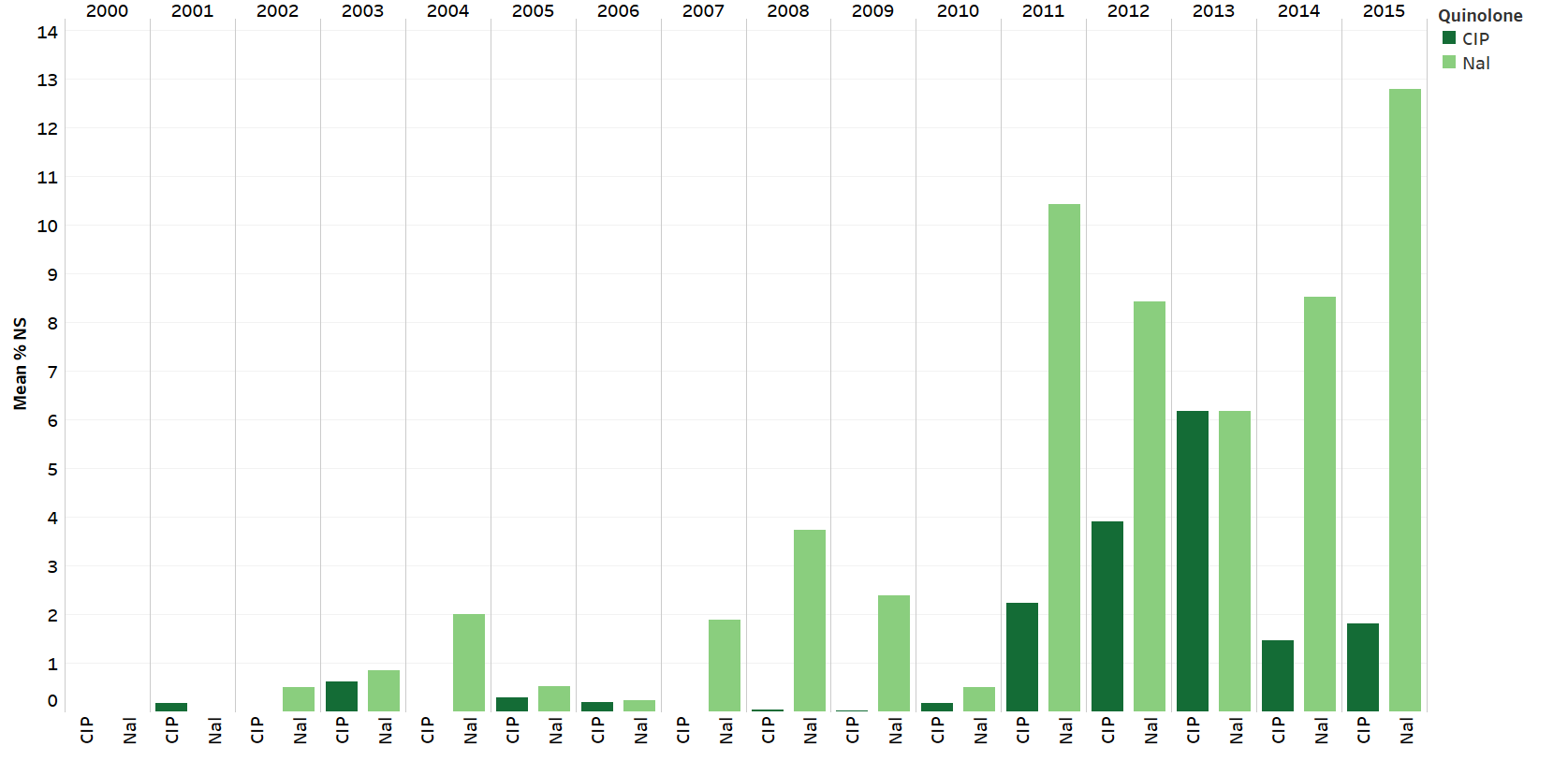

Supplement: S3 Fig — ReLAVRA = La Red Latinoamericana de Vigilancia de la Resistencia a los Antimicrobianos. (TIF) [file pone.0220445.s003.tif]

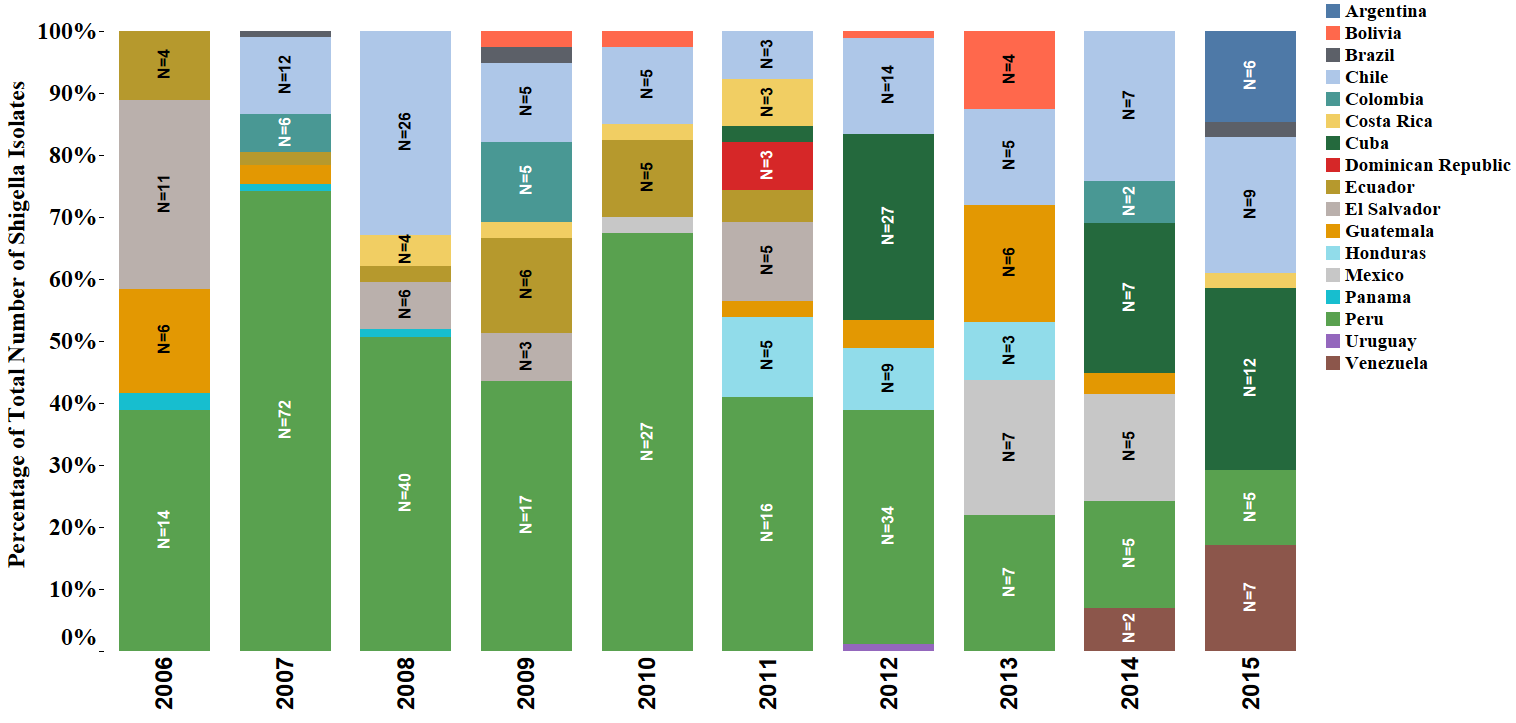

Supplement: S4 Fig — ReLAVRA = La Red Latinoamericana de Vigilancia de la Resistencia a los Antimicrobianos. N = number of isolates reported. (TIF) [file pone.0220445.s004.tif]

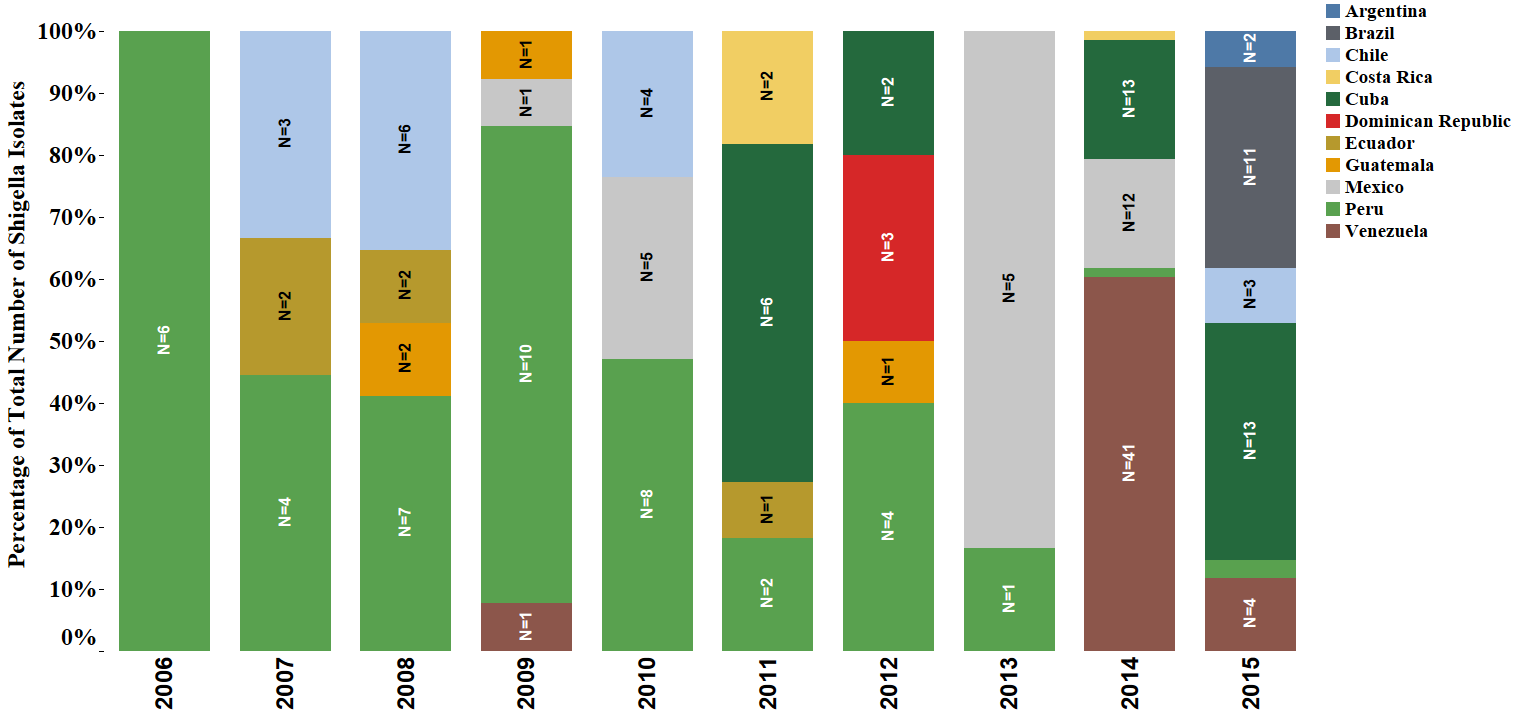

Supplement: S5 Fig — ReLAVRA = La Red Latinoamericana de Vigilancia de la Resistencia a los Antimicrobianos. N = number of isolates reported. (TIF) [file pone.0220445.s005.tif]
